# A review of factors affecting the transfer of sexual and reproductive health training into practice in low and lower-middle income country humanitarian settings

**DOI:** 10.1186/s13031-017-0118-9

**Published:** 2017-09-01

**Authors:** Kristen Beek, Angela Dawson, Anna Whelan

**Affiliations:** The Australian Centre for Public and Population Health Research, Faculty of Health, University of Technology, Sydney, Level 7, 235 Jones St, PO Box 123, Ultimo, NSW 2007 Australia

## Abstract

**Background:**

A lack of access to sexual and reproductive health (SRH) care is the leading cause of morbidity and mortality among displaced women and girls of reproductive age. Efforts to address this public health emergency in humanitarian settings have included the widespread delivery of training programmes to address gaps in health worker capacity for SRH. There remains a lack of data on the factors which may affect the ability of health workers to apply SRH knowledge and skills gained through training programmes in humanitarian contexts.

**Methods:**

We searched four electronic databases and ten key organizations’ websites to locate literature on SRH training for humanitarian settings in low and lower-middle income countries. Papers were examined using content analysis to identify factors which contribute to health workers’ capacity to transfer SRH knowledge, skills and attitudes learned in training into practice in humanitarian settings.

**Results:**

Seven studies were included in this review. Six research papers focused on the response stage of humanitarian crises and five papers featured the disaster context of conflict. A range of SRH components were addressed including maternal, newborn health and sexual violence. The review identified factors, including appropriate resourcing, organisational support and confidence in health care workers that were found to facilitate the transfer of learning. The findings suggest the presence of factors that moderate the transfer of training at the individual, training, organisational, socio-cultural, political and health system levels.

**Conclusion:**

Supportive strategies are necessary to best assist trainees to apply newly acquired knowledge and skills in their work settings. These interventions must address factors that moderate the success of learning transfer. Findings from this review suggest that these are related to the individual trainee, the training program itself and the workplace as well as the broader environmental context. Organisations which provide SRH training for humanitarian emergencies should work to identify the system of moderating factors that affect training transfer in their setting and employ evidence-based strategies to ameliorate these.

## Background

A lack of access to sexual and reproductive health (SRH) care is the leading cause of morbidity and mortality amongst displaced women and girls of reproductive age [1]. Maternal and new-born health (MNH) are threatened by displacement and interruption to health services. Vulnerability to sexual and gender-based violence (SV/GBV) can increase in the aftermath of natural disasters, in conflict settings and as a result of forced migration. Social instability, trauma, economic vulnerability, a lack of education and work opportunities, and the disruption of family planning and medical treatment and prevention services are recognised as important risk factors for the transmission of HIV and other sexually transmitted infections (STIs) [2–4]. In 2015, an estimated 65.3 million people were forcibly displaced worldwide [5], and globally, more than 125 million people are currently in need of humanitarian assistance due to conflict, natural disasters or other hazards [6]. The SRH needs of these displaced populations in humanitarian settings is of concern, including the need to ensure competent assistance to prevent “death, disease and disability related to unwanted pregnancy, obstetric complications, sexual and other forms of gender-based violence, HIV infection and a range of reproductive disorders” [7].

There has been growing global acknowledgement of the importance of addressing the SRH needs of people affected by crises. The work of organisations such as the Inter-agency Working Group on Reproductive Health in Crises (IAWG) has resulted in the development of the Minimum Initial Service Package (MISP). This package involves a standard approach to the coordination of SRH resources and services in humanitarian settings, including the delivery of interventions to address sexual violence; HIV/other STIs; and maternal and new born health in the emergency response phase, and planning for comprehensive SRH services as the situation allows. A 2004 inter-agency global evaluation of reproductive health services for refugees and internally displaced persons [8] found, amongst other challenges, a lack of responders who were adequately qualified or trained to implement components of the MISP. Since the release of this evaluation there has been on-going global investment into building the capacity of international and local health care workers to better provide quality clinical SRH services that are well coordinated and managed. While more recent assessments conducted by IAWG [9] have confirmed that improvements are being made, the development of individual and collaborative capacity remains a key challenge to delivering the life-saving services and activities provided for in the MISP.

A range of SRH short courses and online modules [10], competency based clinical trainings (such as those provided by the RAISE Initiative), and programme specific trainings and workshops have been developed for health workers in humanitarian settings. These courses cover a range of aspects of SRH in the fields of humanitarian preparedness and response. Such opportunities for education and training are crucial components of capacity building and health system strengthening, as without motivated and competent health workers, evidence based interventions cannot be delivered in crisis settings [11]. However, the availability of curricula, provision of training courses and increased numbers of health workers trained do not necessarily indicate that capacity has been built, or that sexual and reproductive health outcomes for crisis affected people have improved. The implementation of training programmes is of little value if the knowledge, skills, and behaviours health workers and managers develop through training are not appropriately applied to the work context and maintained over time [12].

Participation in a training program and the demonstration of knowledge and skills learned as a result are only the first steps towards the practical application of learning. A number of factors moderate the ability of a trainee to transfer newly acquired knowledge, skills, attitudes and behaviours into practice [13–15]. These potential moderating factors may concern the individual trainee, be related to the training program, the organisation, or the context in which the trained individual is working, and can influence how and if trainees are willing or able to use what they have learned at work. An understanding of the factors which impede or facilitate the transfer of training is important as these moderators may be addressed through targeted interventions before, during and after training to maximise the transfer of learning into practice in the workplace, including in humanitarian settings [14, 16, 17].

Research is available concerning effective methods to train health workers for humanitarian response [18]. However, we were unable to locate any reviews that provide evidence concerning the transfer of SRH training in humanitarian settings for in-country health workers working in LMIC. This is problematic considering the large financial investment and political commitment made to building health workers’ capacity in humanitarian settings. Scaling up efficacious training of in-country health workers is of critical importance as these professionals are the first and often best placed responders to deliver accessible life-saving SRH services in disaster contexts. There remains an important knowledge gap between reports of training activities undertaken in this sphere and evidence of outcomes, in particular evidence that provides an understanding of why trainees do and do not, or can and cannot make use of such training on their return to work. Such insights are critical to the design of training programmes and supportive environments that can maximise health worker performance. The first step to improving transfer involves identifying those factors which work for or against the application of knowledge and skills in practice [15]. These factors may also have relevance to the utilisation of training in development contexts, but our review suggests that they are influential and even less well understood in humanitarian settings. The purpose of this paper, therefore, is to review evidence from the literature to determine which factors influence the transfer of training on SRH in humanitarian settings.

## Methods

### Search protocol

An initial scoping exercise identified databases and websites where literature on SRH in humanitarian settings could be retrieved, and assisted with the selection of potential keywords. Four electronic databases (PubMed, Scopus, ProQuest Health and Medical Complete and Medline), websites of 10 relevant organisations (IAWG, Reproductive Health Response in Crisis Consortium, Women’s Refugee Commission, RAISE Initiative, Marie Stopes International, JSI Research and Training Institute, Jhpiego, United Nations Population Fund, World Health Organisation, and International Federation of Red Cross and Red Crescent Societies), and reference lists of key documents were searched to retrieve relevant citations. In this search, the following terms were employed: ‘training’ and ‘reproductive’ and/or ‘sexual’ (health) and ‘humanitarian’; ‘capacity’ and ‘building’ and ‘reproductive’ and/or ‘sexual’ (health) and ‘humanitarian’; ‘capacity’ and ‘reproductive’ and/or ‘sexual’ (health) and ‘humanitarian’; ‘training’ and ‘health’ and humanitarian’; and ‘capacity’ and ‘health’ and ‘humanitarian’. Electronic searches were limited to only those in the English language and those falling within the contemporary timeframe of January 2004–December 2014.

### Study selection and quality appraisal

A Population, Interventions, Comparators, Outcomes, Study design (PICOS) question was formulated to guide this review. As per guidelines, a PICOS question may be used to identify specific evidence to inform clinical or health service practice [19]. The PICOS question for this study was: For health workers from LMIC, what factors support or constrain the application of learning gained from training on SRH in humanitarian settings?

SRH activities, services and resources are defined as outlined in the Inter-agency Field Manual on Reproductive Health in Humanitarian Settings [7]. This includes both the MISP and those components of comprehensive SRH which are to be built upon the MISP as the situation allows. The term humanitarian setting is used to pertain to any hazard: natural, armed conflict, complex emergencies, political repression, epidemics, or technological [20], and resulting crises of any type or scale in any LMIC context. A diversity of research evidence was sought for this study and therefore papers using qualitative, quantitative and mixed methodological approaches were deemed suitable for inclusion.

The inclusion/exclusion criteria used to select papers in this review is presented at Table 1.

**Table 1:** Inclusion/exclusion criteria used in this review

Included	Excluded
In English	In languages other than English
2004–2015	Pre 2004
Papers pertaining to training on any component of sexual and/or reproductive health outlined in the Inter-agency Field Manual on Reproductive Health in Humanitarian Settings, clinical and/or non-clinical	Training on general health
Description of the training program and a discussion of factors which facilitated or impeded the transfer of training into practice	Training and transfer process not described, training recommended not conducted
Papers addressing any point in the continuum of an emergency- from mitigation and preparedness, through response in the acute phase, post-emergency and protracted disaster response, to building more durable solutions	Development settings
LMIC contexts	High Income Country contexts
In-service or continuing professional development training courses, workshops, exercises/simulations, continuing medical education, multi-media training	Pre-service training, pre-deployment training for expatriate health personnel, self-directed learning, use of guidelines independent of training
In-country health workers	Expatriate/internationally deployed health workers
Established in-country health workers (any cadre) with some prior health training (not restricted to SRH or disaster health) and experience	Newly trained health workers (any cadre)

**Fig. 1:**
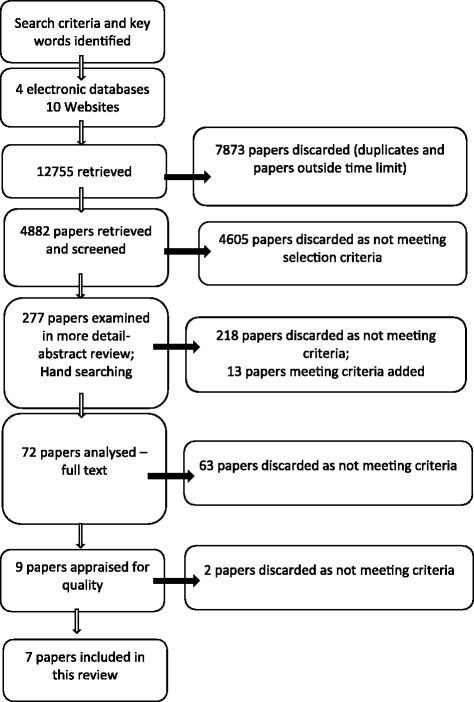
Literature Review Process

In accordance with the above selection criteria, the literature review process followed the guidelines outlined in the Preferred Reporting Items for Systematic Reviews and Meta-Analyses (PRISMA) [21] (see Fig. 1).

The literature search identified 12,755 potentially relevant documents. Of these, 7873 were discarded as duplicates, or outside the 2004–2014 timeframe. The remaining 4882 papers were screened, and 4605 excluded as they did not meet selection criteria. Of the remaining 277 citations, 218 were excluded by abstract review. The reference lists of these 227 papers were reviewed and 13 potentially relevant papers added. An analysis of the full-text of the papers was conducted on 72 citations, of which 63 were excluded as they did not help to answer the PICOS question, were concerned with high income countries, general health training, pre-service education, or failed to meet the inclusion criteria in another way. Nine papers were found to be relevant in understanding factors which affect the ability of in-country health workers to apply their SRH training in humanitarian settings. These nine papers were then appraised for quality using the Critical Appraisal Skills Programme (CASP) assessment tool [22] and Pluye et al.’s (2009) scoring system for appraising mixed methods research [23]. After discarding two papers that did not meet quality appraisal criteria, 7 documents remained and are included in this review.

### Data abstraction and synthesis

A content analysis methodology was applied to the remaining papers in order to categorise and count categories in a systematic and replicable manner [24]. This was guided by a grouping of factors affecting the transfer of training derived from the literature on the topic. Training transfer is a widely studied area of research within the human resources, education and psychology fields. There has been a proliferation of conceptual and research-based work on understanding the system of factors which may contribute to or relieve the “transfer problem” [25] of training programmes [13]. A recent qualitative review by Burke and Hutchins [14], systematic review by Blume et al. [13] and review by Grossman and Salas [17] have synthesised evidence on which potential moderating factors, working before, during and after training, impact on the transfer of training to different contexts and tasks. In this literature, factors which influence the transfer of training are generally organised on the three levels of individual trainee, training programme, and organisation, and include such considerations as an individual’s self-efficacy, motivation, organisational commitment, job involvement, learning goal orientation, voluntary participation in training, and post-training knowledge; the relevance of a specific training programme’s contents to the trainee and their workplace needs, and the way in which the training is conducted and followed-up; and resourcing and support within the organisational environment. The influence of these training transfer factors may be positive or negative. For example, organisational support may be strong for the transfer behaviour, thus acting as an enabler to transfer. Alternatively, a trainee may perceive little relevance of the training tasks to their daily work, and as such, the transfer factor of content relevance would work to undermine the transfer of newly developed skills to the workplace. Taken together, individual, training and organisational training transfer factors have been shown to explain why training may or may not be transferred to practical use in work settings.

The analysis of the findings sections of papers under review involved the identification of factors known to impact the transfer of knowledge, skills and attitudes learned in training courses into practice. However, evidence of further moderating factors not discussed in the training transfer literature were also sought.

Tables were created to explore the identified factors and examine patterns across the research. These were discussed by authors and consensus reached on the factors identified, as well as the gaps.

## Results

Seven papers were included in this study. The characteristics of documents under review are outlined in Table 2.

**Table 2:** Characteristics of documents included in review

Reference	Method	SRH Area	Training Participants	Disaster Type	Research Aim
Homan FF., et al. (2010) [34].	Quantitative	MNH	Clinicians	Conflict	To present results of a project to establish primary healthcare-based antenatal care in post-conflict Kosovo.
Nelson BD., et al. (2012) [26].	Mixed Methods	Maternal, newborn and child health	Frontline Health Workers (FHWs) (mixed group, partially meeting review inclusion criteria)	Conflict	To develop, implement and evaluate a novel training package on maternal, newborn and child survival for frontline health workers in South Sudan.
Olness, K., et al. (2005) [27].	Mixed Methods	Broad components including MNH & S/GBV	Physicians and health care professionals	All	To review experiences in designing and implementing a training course for international health care professionals in disaster management focussed on the needs of children.
Smith, J. R., et al. (2013) [28].	Mixed Methods	SV	Healthcare Providers (HCPs)	Diverse	To evaluate the effect of clinical care for sexual assault survivors multimedia training on healthcare providers’ attitudes, knowledge, confidence and practices in 4 countries.
Sullivan, T. M., et al. (2004) [33].	Mixed Methods	MNH, post-abortion care, infection prevention.	Clinicians	Conflict	To present results and lessons learned on a project to use evidence to improve reproductive health quality along the Thailand-Burma border.
Tanabe, M., et al. (2013) [29].	Qualitative	SV	Community Health Workers (CHWs) (included in this review); Traditional Birth Attendants (not included in this review).	Conflict	To examine the safety and feasibility of community-based care for sexual assault survivors to contribute to building evidence on alternative models of care in humanitarian contexts.
Teela, K. C., et al. (2009). 68(7): 1332–1340 [31].	Qualitative	MNH	Maternal Health Workers (MHWs)	Conflict	To provide perspectives of maternal health workers on community-based delivery of maternal care in conflict-affected eastern Burma.

A range of transfer moderating factors that are well described in the training transfer literature were identified in the included papers. In addition to this, factors pertaining to the broader socio-cultural and political environment were described in the included studies. As a result, a broader ‘environmental’ level may be added to the factors known to influence the ability of health workers to transfer knowledge and skills in the unique context of SRH in humanitarian crises. Pertinent training transfer factors are described below.

### Individual level factors

#### Post-training knowledge

In order for the transfer of training to occur, participants must learn and be able to retrieve new knowledge and apply skills. Of the seven studies included in this review, four papers provide details on the extent of learning which occurred as a result of undertaking the training [26–29]. Tanabe et al. (2013) showed that community health workers (CHWs) from pilot sites were knowledgeable about the use of required clinical skills for sexual assault survivors, the need for confidentiality and the use of forms and information management processes. Olness (et al. 2005) used pre- and post- tests to demonstrate that significant knowledge improvements had been achieved in all countries except one (which had higher pre-test results).

Smith (et al. 2013) found that a multimedia training tool on care for sexual assault survivors was effective in improving health care providers’ (HCPs) “respect for patient rights, knowledge, confidence, and clinical practice” (p10), but did not change negative attitudes such as blame and disbelief three months after the course had been delivered. Nelson et al. (2012) found an increase in knowledge and skills amongst frontline health workers (FHWs) pre- and immediately post-training, but noted a significant decline in skills two to three months post-training, particularly for tasks that were not previously part of skillsets.

#### Self-efficacy

Self-efficacy is a concept founded in social learning theory which generally refers to beliefs or judgements people, in this case trainees, make about their “capabilities to organise and perform the courses of action needed to achieve given goals” [30]. Self-efficacy has been found to influence the transfer of training [13], and while the term encompasses a broad set of factors, only the related concept of ‘confidence’ was identified in four of the included papers [27, 28, 29, 31]. In their evaluation of a training program, which included sessions on emergency obstetrics, newborn resuscitation and sexual and gender based violence, Olness et al. (2005) found positive trainee responses to questions of confidence in fulfilling all learning objectives at the conclusion of the course. In work by Smith et al. (2013), confidence in the use of clinical skills for the care of sexual assault survivors in humanitarian settings was found to differ by sex, trainee profession and prior experience. Teela et al. (2009) found differences in levels of confidence to be based on content area covered in the training course, but that “increased ownership over the project…, regular training and capacity-building workshops, and practical experience” (p1337) led to increased confidence among maternal health workers (MHWs). In a similar way, Tanabe et al. (2013) report that CHWs were generally confident and comfortable with the subject matter of the training, but that this confidence declined when asked about implementing new skills such as history-taking and psychosocial care.

#### Motivation to transfer

Motivation to transfer training includes those processes which influence “the intensity and persistence of efforts that trainees apply in learning-oriented improvement activities” [14], and has been identified as an antecedent to transfer. While no study included in this review directly mentions ‘motivation’, related concepts were presented in two studies [29, 31]. Tanabe et al. (2013) report the ‘enthusiasm’ expressed by training course participants about the opportunity to provide needed care in their communities. Researchers linked this level of enthusiasm to “the utility and promises of this community-based approach in settings where insecurity and other barriers prevent access to facility-based care” (p10). Similarly, Teela et al. (2009) found that the ‘pride’ expressed by MHWs was related to their ability to provide “pragmatic solidarity” or a useful service to their communities in times of crisis.

### Training level factors

#### Content relevance

The alignment between a training programme’s contents and objectives and a trainee’s perceptions of applicability to job requirements has been shown to correlate with training transfer. Three studies included in this review discuss the importance of providing training which is relevant and responsive to trainee and community needs [26, 29, 31]. Teela et al. (2009) found that continuous discussion with MHWs helped to identify important gaps in the initial training and areas which required follow-up training, allowing project implementers to develop subsequent workshops.

Work by Nelson et al. (2012) showed the importance of providing training content and materials pitched at the correct level for trainees, in this case, wholly pictorial materials to their frequently non-literate trainee audience. Tanabe et al. (2013) reported that the training CHWs received on community-based care for survivors of sexual assault did not directly address intimate partner violence- the most common form of gender-based violence in this setting. This brought into question the completeness of training content to trainees, who consequently “expressed interest in learning more about domestic violence and stated it as an important issue for community awareness” (p9).

### Follow-up

Follow-up attention and activities initiated by supervisors and training providers have been shown to increase the long-term use of trained skills [14]. The studies by Smith (et al. 2013), Sullivan et al. (2004) and Teela (et al. 2009) highlight the importance of providing follow-up and regular refresher trainings. The first study indicates that the most significant changes to HCPs’ attitudes towards care for sexual assault survivors was found when case managers followed up after training. Similarly, improvements in quality of care gained as a result of the project studied by Sullivan et al. (2004) were sustained through weekly follow up quality assurance meetings. Research by Teela (et al. 2009) linked regular refresher training and workshops to an increase in confidence amongst MHWs.

### Organisation/project level factors

#### Transfer climate: Workplace cues

The transfer climate in which a trainee will attempt to apply their new skills or knowledge has been shown to include observable or perceived workplace factors that may support or hinder the use of these new capacities [32]. Within this climate, training transfer research highlights the importance of situation cues within a broader set of strategies to prompt trainees to use new skills. These cues may include trainee reflective practices, and the implementation, design or provision of informational, procedural or decision making aides [17]. The importance of supporting the use of training with job aides such as checklists and forms to cue and guide practice was highlighted by three papers included in the review [26, 28, 33]. Nelson et al. (2012) credit the success of training on maternal, newborn and child health, in part, to the provision of setting-appropriate checklists as part of a comprehensive ‘training package’. FHWs reported that these were “useful visual reminders of when to refer patients to higher level care” (p133) and to educate women about the importance of prenatal care. Sullivan et al. (2004) credit the addition of checklists to client record cards to improvements in information provided to clients of a reproductive health clinic on the Thailand-Burma border. Smith et al. (2013), highlight the need for job aides such as history and exam forms, and written drug treatment protocols to prompt correct practice in response to their findings that less than one-third of health care provider trainees could recall certain procedures 3 months post-training.

#### Transfer climate: Resources

Resourcing, which may include budgetary, logistical or practical support, was frequently identified as a potential hindrance to the use of trained skills in work contexts. The study by Nelson et al. (2012) recognised that trainees cannot transfer trained skills without the requisite resources which enable them to do so. In response, and due to the limited supply lines in South Sudan, it was found necessary to “directly equip each [FHW] with reusable and setting-appropriate equipment” (p134).

The study by Sullivan et al. (2004) into improving reproductive health quality along the Thailand-Burma border found through facility audit that it was necessary to transform examination spaces for privacy and provide sinks for handwashing in or near consultation areas to support training on universal precautions. Likewise, Homan et al. (2010) report that implementing partners supplied primary health care facilities involved in training staff on antenatal care with an implementation package or “toolkit” of resources necessary for the use of newly trained skills in health centres.

#### Support

Support from supervisors was found to be a significant factor enabling health workers to put their learning into practice. Smith et al. (2013) found that “[o]rganisational and contextual factors influenced uptake of the training. The most significant improvements to HCPs’ [health care providers’] attitudes were demonstrated in Kenya and Ethiopia, where gender-based violence case managers participated in training” (p9). The importance of support also extended to the organisation more generally, as findings showed “[i]nstitutional involvement and commitment has been established as a key element to ensuring facility-wide preparedness to respond to sexual violence” (Ibid). Likewise, Sullivan et al. (2004) note the importance of a system of supportive supervision and mentoring in order to enhance and sustain skills.

### Additional factors

In addition to factors which fall within the traditional categories of individual, training or organisational, this review found that a broader level of factors may function as barriers or facilitators to the use of new skills and knowledge in this field.We have categorised these as ‘environmental level’ factors as they operate above and around the learner, training intervention and organisational factors.

### Society and culture

The existing socio-cultural norms and mores of potential beneficiaries and the trainees themselves were found to be potential moderators of training transfer. Amongst these, the trust and/or confidence potential clients felt towards the offered services were found to significantly influence the application of newly trained knowledge and skills in three of the seven papers included in this review [29, 31, 34]. In describing the post-Soviet health care system of Kosovo as ‘specialist-oriented’, Homan et al. (2010) remark that “changing the cultural view [of patients] to have confidence in family medicine and primary care will take time” (pp32–33).

The issue of trust was found to extend more specifically to HCWs and the services being offered in studies by Teela et al. (2009) and Tanabe et al. (2013). Findings from eastern Burma show that “a community or client’s trust in a program or its workers influences service-use” [34: p1335]. Researchers here found that the development of cooperative and trusting relationships between newly trained service providers and the community facilitated acceptance and implementation.

Similarly, Tanabe et al. (2013) state that “community members need to feel comfortable in seeking care” (p8) and suggest additional training on elements such as counselling, listening and empathy to enhance trust within the community. Related to trust and confidence is the belief in a HCWs’ technical competence to provide the services offered. This was identified as an important factor by Teela et al. (2009) who found that demonstration of clinical skills was critical to acceptability and the provision of timely care. CHWs in the study by Tanabe et al. (2013) also expressed the importance of reminding community members that, along with confidentiality, “we are capable to provide this care and have been trained to do so” (p7).

Traditional beliefs and practices which could impede the transfer of trained skills were also noted. This was seen by Teela et al. (2009) in the position MHWs felt they held in the community relative to that of traditional community providers, who were more likely to be trusted and therefore approached for services. The authors of this study also noted an incident where “the family of a woman who required an urgent blood transfusion insisted on first performing a traditional religious ceremony with a healer” (p1335). In highlighting these cases, Teela et al. (2009) suggest that if newly trained MHWs can build positive relationships with traditional community providers through partnership and respect, communication links between health workers of all types and between health workers and the community may be improved.

Tanabe et al. (2013) discuss cultural stigma regarding SGBV. The authors note that the cultural responses of “shyness; fear of others’ opinions, such as the scolding of parents or the fear of being looked down upon by community members; shame; and concerns that they may not receive help” (p8) are important barriers to the willingness of sexual assault survivors to seek care. They suggest that these factors could be overcome with increased outreach and awareness raising activities in settings where newly trained CHWs are placed. In a similar way, Teela et al. (2009) state that “a more refined framework for achieving improved access within a community-based program should consider other factors such as social norms surrounding care-seeking, perceptions of the seriousness of obstetric emergencies, gender and power-relations, household-decision-making, and traditional practices” (p1338).

### Health system factors

Aspects of the health system in which newly trained health workers are to work, including policy, financing and capacity, were found to be significant in five of the papers included in this review. Smith et al. (2013) explain the importance of policies and protocols to the successful use of newly trained skills in practice, stating that for trainees to effectively provide clinical care for survivors of sexual assault “national or international protocols must be easily available in all facilities in the language of the provider and all providers should be made aware of their importance” (p9). A further health governance issue was reported by Smith et al. (2013) in their finding that policy restrictions on the availability of emergency contraception remained an obstacle to the use of trained skills in an urban refugee setting in Jordan (p9).

A lack of adequate and appropriate financing, physical infrastructure and resourcing was identified as an important factor in four of the included papers. Homan et al. (2010) count damaged infrastructure, resources and funding as among the “innumerable challenges” (p32) faced in establishing antenatal care at a primary health care level in post-conflict Kosovo. Smith et al. (2013) report “health facility level barriers including stock-outs of HIV [post-exposure prophylaxis]” (p9) for clinical care of sexual assault survivors in Democratic Republic of Congo. More specifically, Teela et al. (2009) detail the disruption to and deliberate destruction of infrastructure, resources and supply chains in active conflict areas in eastern Burma.

In terms of health workforce, three of the papers included in this review emphasise the importance of collaboration and integrating newly trained staff and/or their skills into health system structures. Olness et al. (2005) predict one of the challenges to allowing trainees to use trained skills will be “in keeping local disaster agencies aware of the local expertise [in the special needs of children in the management of disasters] available to them” (p247). Nelson et al. (2012) explain that one potential explanation for the success of their training programme was that “the initiative was greatly facilitated through close collaboration with federal and state ministries of health and nongovernmental partners” (p134). Collaboration between tiers of health workers was found to be an important contributor to programme success by Teela et al. (2009).

### Politics

Political uncertainty and insecurity were regarded as important factors for the implementation of trained skills in two of the papers [31, 34]. Teela et al. (2009) note specific threats to MHWs due to their ethnic affiliations and the deliberate targeting of health workers and their resources. More indirectly, Kosovo’s position at a transition point between humanitarian relief and longer-term development, and the political uncertainty inherent in a newly founded nation are listed by Homan et al. (2010) as a challenge to implementation.

### Physical setting

Finally, Teela et al. (2009) discuss the important logistical constraints of difficult terrain and climatic factors. Target populations were often accessible only by walking for long periods through difficult conditions, and this proved challenging for MHWs in applying newly acquired skills into practice. In this case, trainees suggested numerous practical solutions at follow-up, and while some were adopted, others, such as the use of donkeys for transport and the passage of supplies, were not due to financial and procurement constraints.

## Discussion

The aim of this review was to synthesise the literature to gain insights into factors which influence the ability of health workers in LMIC to transfer SRH training into practice in humanitarian settings. To our understanding, this is the first review to extract meaningful lessons from the published literature on why health workers do or do not use newly trained skills and knowledge to improve SRH for people in emergency situations.

The limited number of research papers identified for this review highlights significant gaps in knowledge. No citations were found which detailed training interventions specifically for mitigation and preparedness. Training for response predominated and three of the seven papers had protracted crises as the setting for response. Conflict was the hazard most commonly discussed, and while the training interventions detailed in two of the references could be applied to diverse or all disaster types, no citations could be found on the specific challenges of disasters resulting from natural, political, complex, epidemic, or technological hazards. Further gaps in the literature concern components of SRH services for humanitarian settings as outlined by the Inter-agency Field Manual on Reproductive Health in Humanitarian Settings [7]. No documents were found in relation to HIV and other STIs, adolescent sexual and reproductive health, family planning, comprehensive abortion care, coordination of an SRH response in the acute phase, and planning for comprehensive SRH services as the situation allows. These gaps in the literature constrain our understanding of which training methods and supportive mechanisms best leverage transfer for different SRH components in diverse humanitarian contexts. Increased documentation including the publishing of research and evaluation findings of organisations involved in this field would be of great benefit to professionals engaged in developing the capacity of humanitarian health workers in SRH.

The findings of this review indicate the influence of a number of important training transfer moderating factors that concur with the training transfer literature, such as the importance of a supportive transfer climate, follow-up and trainee perceptions of self-efficacy. However, included studies only tangentially consider transfer, and no study specifically sought to understand the full system of potential training transfer moderating factors that may be unique to SRH in humanitarian settings. This could point to a lack of attention to transfer in the development and evaluation of training programmes in this field.

The finding of additional moderators in this review, which we have labelled ‘environmental level’ factors, have implications for the training transfer literature as it relates to training for SRH in humanitarian settings, and the development of capacity among humanitarian staff more generally. Socio-cultural factors, health system capacity issues, the political environment and the physical setting of transfer were found to have important mediating relationships with transfer in a majority of the studies included in this review. This warrants further investigation and may require consideration of additional frameworks and conceptual approaches, such as health systems strengthening [31] and the social determinants of health [32] to better understand the dynamics at play between training and training transfer in these particular settings.

An important contribution of this review is the finding that, while training can be an effective strategy to improve knowledge and confidence, ensuring that new learning is maintained over time, or that gains in knowledge and skills will be transferred to the workplace is complex and multifactorial. Successes documented in the studies included in this review almost invariably relied on scaffolding strategies to facilitate transfer. For example, post-training improvements in knowledge were maintained and gaps in understanding addressed through regular follow-up and refresher trainings [27, 28, 29]. Supportive transfer climates were ensured through adequate resourcing, encouraging support and providing cues to prompt the use of new skills [27, 28, 34]. Sociocultural issues of trust and confidence were addressed through strategies of building respectful cooperative relationships between the programme, community members and tiers of health workers [29, 32, 35].

This review highlights the importance of identifying potential transfer moderating factors on multiple levels during the design or pilot phase, and integrating strategies to either harness or ameliorate these moderators before, during and in follow-up to the training programme. Each potential moderating factor identified may be seen as a point of intervention to maximise transfer and a component to be included during the evaluation of training. A key lesson here is that training should not be implemented as a stand-alone strategy without consideration of the system of moderating factors which operate to influence its effectiveness. As Homan et al. (2010) explain, “[i]t is all too common for international aid initiatives to focus too narrowly on training or resource distribution, without adequate attention to improving the systems that are required for ongoing success” (p32).

Some domains of moderating factors may be more amenable to intervention than others and implementing interventions to address factors at the environmental level may prove difficult and require the addition of strategies which lie outside those of a traditional training programme. Advocacy, community awareness raising, health systems strengthening and rights-based approaches may be useful in producing an environment which supports the use of newly trained skills. While this work may not be so straightforward, findings from this review suggest that ignoring the influence of environmental level factors could compromise the use of training and therefore the effectiveness of training programmes. In the field of humanitarian response, where the call for training to build capacity is very often repeated, this may require hard decisions. Training may be seen as a direct way to develop the competencies of health workers to respond to health needs in humanitarian crises, but without an environment supportive of the use of this training, scarce aid resources may be wasted.

### Limitations

This review may have been limited by an incomplete retrieval of relevant papers, though efforts were made to use appropriate databases and the resource/reference lists on websites of the most pertinent organisations to this field of study. An important limitation of this work is in the small number of studies meeting inclusion criteria and the content gaps on training type, disaster and humanitarian setting typologies, phase of humanitarian response, SRH subject matter covered by training and training type. This paper has provided preliminary insights into factors which may intercede between training and transfer. However, future research should also address how the moderating factor- transfer relationship operates in each diverse setting, for differing content areas and training types, and for both facility-and community-based service delivery models. It may also be beneficial to better understand how identified moderating factors interact across and within levels to influence transfer. Strategies which may leverage transfer are suggested by the included literature, but given the limited number of resources found, it is difficult to draw conclusions on which specific interventions may be appropriate in different humanitarian settings, and for different SRH components and cadres of health care workers. Further research could provide more specific insights into how best to maximise transfer in diverse contexts. As discussed above, the transfer of trained knowledge and skills is an important first step for training programme effectiveness, but it does not necessarily guarantee effectiveness in performance. There is therefore opportunity to extend this research longitudinally from use of training to effective use of training [13].

## Conclusion

People, particularly women and girls, need access to appropriate SRH services in humanitarian contexts, where vulnerabilities to related death, disease and disability are often exacerbated. International commitment to developing the capacity of health workers to provide these services is evident and a number of training courses, curricula and capacity building programmes have been implemented. There remain, however, important gaps in our understanding of how health workers can apply knowledge and skills gained through training programs to best provide SRH services in diverse humanitarian settings. Transfer is a crucial first step to training programme effectiveness, and it is necessary to identify potential transfer moderating factors so that they may be addressed during the design, delivery and follow-up phases of training programmes so that transfer may be leveraged. This review has provided some insight into elements which affect the transfer of training in humanitarian health contexts. Buttressing strategies are necessary to assist trainees in using newly acquired knowledge and skills in their work and these supportive interventions must address factors at the individual trainee level, the training curricula and the workplace and wider environment context. Organisations that provide training on SRH for humanitarian emergencies should work to identify the system of moderators particular to their setting and, using this as a diagnostic mechanism, employ a set of strategies which, based upon evidence, can better facilitate the transfer of training. Only when knowledge and skills are appropriately and effectively applied in the field can evidence- based lifesaving SRH care services be provided for women, girls, boys and men in humanitarian settings.

## Abbreviations

CASP: Critical Appraisal Skills Programme
CHW: Community Health Worker
FHW: Frontline Health Worker
GBV: Gender-based Violence
HCP: Healthcare Provider
HIC: High Income Country; HIV Human Immunodeficiency Virus
IAWG: Inter-agency working group on reproductive health in crisis situations
LMIC: Lower Middle Income Country
MHW: Maternal health worker
MISP: Minimum Initial Service Package
MNH: Maternal and Newborn Health
PICOS: Population, Interventions, Comparators, Outcomes, Study
PRISMA: Preferred Reporting Items for Systematic Reviews and Meta-Analyses
SRH: Sexual and reproductive health
STI: Sexually transmitted infection
SV: Sexual Violence
